# Xenomonitoring of sleeping sickness transmission in Campo (Cameroon)

**DOI:** 10.1186/s13071-016-1479-4

**Published:** 2016-04-12

**Authors:** Pascal Grébaut, Trésor Melachio, Simplice Nyangmang, Vincent Ebo’o Eyenga, Guy-Roger Njitchouang, Elvis Ofon, Flobert Njiokou, Gustave Simo

**Affiliations:** UMR177 IRD/CIRAD INTERTRYP, TA A17 G, Campus International de Baillarguet, 34398 Montpellier cedex 5, France; Department of Animal Biology and Physiology, Parasitology and Ecology Laboratory, Faculty of Science, University of Yaoundé I, P.O. Box 812, Yaoundé, Cameroon; Programme National de Lutte contre la Trypanosomiase Humaine Africaine, Ministry of Health, Yaoundé, Cameroon; Center for Research on Filariasis and other Tropical Diseases, P.O. Box 5797, Yaoundé, Cameroon; Molecular Parasitology and Entomology Unit, Department of Biochemistry, Faculty of Science, University of Dschang, P.O. Box 67, Dschang, Cameroon

**Keywords:** Human African trypanosomiasis, Glossina, Xenomonitoring, Transmission, Risk

## Abstract

**Background:**

The sleeping sickness focus of Campo in South Cameroon is still active, at a low endemic level, for more than a century, despite a regular medical surveillance. The present study focuses on the spatial distribution of xenomonitoring information obtained from an entomological survey performed in the dry season 2012. It appears that humans constitute a third of the blood meals and that the flies’ densities were coherent with those classically observed in the different biotopes. Paradoxically, the epicenter of the focus is the place where the risk indicators are the lowest ones.

**Methods:**

Particular attention was paid to the entomological device so that it covered the main part of human activities in the study area. One hundred and sixty-two pyramidal traps were used to catch tsetse flies twice a day that were identified, counted, dissected. Molecular analysis using classical and specific molecular markers was conducted to determine the importance of trypanosome infections and the nature of the feeding hosts. This information was used to calculate a Transmission Risk Index and to define a gradient of risk that was projected into a Geographical Information System.

**Results:**

Conventional entomological indicators such as species identification of tsetse flies or the Apparent Density per Trap per day, show that *Glossina palpalis palpalis* is the main species in the campo area which is classically distributed into the different biotopes of the study area. Molecular analysis reveals that humans constitute a third of the blood feeding hosts and that 20 % of the dissected flies were infected with trypanosomes, principally with *Nannomonas*. Nevertheless, one fly was carrying *Trypanosoma brucei gambiense*, the pathogen agent of sleeping sickness, showing that the reservoir is still active in the epicenter of the focus. Paradoxically, the Transmission Risk Index is not important in the epicenter, demonstrating that endemic events are not only depending on the man/vector contact.

**Conclusion:**

Xenomonitoring provides a valuable guide/tool to determine places at higher risk for vector/human contact and to identify trypanosomes species circulating in the focus. This information from xenomonitoring demonstrates that decision makers should include a veterinary device in a control strategy.

## Background

Since 2010, human African trypanosomiasis (HAT), or sleeping sickness, has been considered by the WHO as a neglected disease to be eliminated within the next decades [1]. In 2012, the number of diagnosed cases fell under eight thousand, reaching the historical level of the decolonization period of the 1960s [2]. While this superficially gives the impression that the disease could soon disappear, several factors still make its elimination a real challenge [3]. First, HAT is considered as a neglected disease, meaning that in most countries where the endemic level is low, few efforts are made to support active screening and vector control in the historic foci. Second, diagnosis and treatment are supplied by specialized medical teams. In most endemic countries, the majority of this staff, which was trained in the 1970s and 1980s, is now beginning to retire. Finally, the perpetuation of an active reservoir (for more than one century in some foci) is critical to the reemergence of the disease [3]. The sleeping sickness focus of Campo, located in southwestern Cameroon, is characteristic of this situation.

The history of this sleeping sickness focus has previously been described [4]. In summary, German colonizers in the beginning of the twentieth century already considered the region of Campo to be dangerous [5]. After the First World War, French colonizers continued to describe an endemic situation in the region. Specifically, a low prevalence was reported, principally concerning the villages of Ipono, Mabiogo and Campo Beach [6]. In 1974, Eouzan and colleagues performed an unpublished entomological survey in Campo, in order to prepare for vector control activities. According to yearly reports published between 1977 and 1997 by the “Organisation de Coordination contre les Endémies en Afrique Centrale” (OCEAC), 146 sleeping sickness patients were passively diagnosed in the Campo area. In 1980, Eteme and Chauvet (unpublished data) proposed a vector control campaign, and insecticide was sprayed twice per year between 1981 and 1986. In 1998, the OCEAC performed a mass screening in the focus [4], which included the Equatorial Guinean population living across the Ntem River, a natural border between the two countries. At that time, 16 cases were diagnosed out of 5,660 screened persons.

Investigating the vector situation, Morlais et al. [7] found that 15.6 % of tsetse flies were infected with trypanosomes in the area. In the following year, isoenzyme analysis allowed the characterization of *T. b. gambiense* in a pig [8]. Cameroonian scientists carried out several studies in this HAT focus between 2000 and 2010, in an effort to understand how an active reservoir is maintained. Njiokou et al. [9] observed a 25.3 % trypanosome infection rate in wild mammals using molecular markers, although 11.7 % displayed unidentified trypanosome species. Subsequently, Simo et al. [10], using a Mobile Genetic Element analysis (MGE-PCR), suggested a flow of trypanosome genotypes between humans and pigs in this focus. One animal was diagnosed positive for *T. b. gambiense* (resulting in a 0.6 % prevalence in wild mammals), whereas 2.6 % were positive for *T. b. gambiense* non-group 1, for which human infectivity has not been established, so it can be considered as *T. b. brucei*. By studying the blood meals of *Glossina palpalis*, Simo et al. [11] determined that 61.4 % of the blood meals in the area were taken on humans, providing evidence for an important relationship between humans and the tsetse vector. Continuing from their earlier study on animals, Njiokou et al. [12] determined that 4.83 % of domestic mammals were infected with *T. b. gambiense*, thus demonstrating the possibility of an animal reservoir. Experimental infections conducted by Van Hoof et al. [13] gave evidence of the transmission of *T. b. gambiense* from animal to man, even after several host changes. Penchenier et al. [14] conducted experimental infections on pigs and described a spontaneous cure from *T. b. gambiense* infection in a few months. Regarding the trypanosome infection rates in tsetse flies, Farikou et al. [15] observed that the infection rate of *T. brucei* was similar in all the villages, and that the infection rate of *T. congolense* was significantly greater in the villages closer to the forest, near the eastern part of the area. Mélachio et al. [16] confirmed that the tsetse population was structured in panmictic subpopulations, whose sizes were estimated between 20 and 300 individuals with densities between 120 and 2000 flies/km^2^and a dispersion of adults between 60 and 300 m.

Xenomonitoring is more efficient today, due to the use of molecular tools and gene databases that enable the identification of trypanosomes, as well as the origin of the tsetse blood meal. Furthermore, the recent development of gene databases enables precise identification of feeding hosts. This type of monitoring has been successfully used in Kinshasa [17, 18] and in Cameroon [15, 19]. In the present study, we have developed a projection of the epidemiological data in order to obtain a spatial representation of HAT in this focus, which comprises both human cases and entomological information. An entomological study was conducted in the Campo focus during the 2012 dry season, yielding a refined view of the distribution of the pathogen actors involved in HAT. The results effectively use xenomonitoring to provide much-needed information that can help to eliminate sleeping sickness from this area.

## Methods

### Study zone

Campo, a small town of about 2,000 inhabitants, is located in the extreme southwestern corner of Cameroon (2°22 N, 9°49E) along the Atlantic Ocean and the mouth of the Ntem River that separates Cameroon from Equatorial Guinea. The environment is composed of a coastal plain along the ocean, mangrove swamp along the Ntem River near the southern part of the area, and evergreen forest. The study zone included Campo and four villages located to the east of the city. Three rivers feed the Ntem watershed in this area: the Bitandé, Nyamelandé and Bibabimvoto rivers. The region has an equatorial climate of the Guinean type, with four seasons per year. Annual changes in temperature are low and the medium temperature is 25.7 °C, with an average relative humidity of 87 % and an annual rainfall greater than 2,700 mm^3^.

Population density is low, with less than one inhabitant per square kilometer. Most people belong to one of four ethnic groups (Yassa, Mvae, Mabea and Pygmies). The habitat is linear, and water points are for public use. Both maritime and inland fishing are practiced, and with the exception of shrimp fishing, are exclusively performed by men. Hunting was previously one of the most practiced activities, but the creation of the Campo Ma’An National Park in 2000 has highly restricted hunting in the area.

### Epidemiological context

**Fig. 1:**
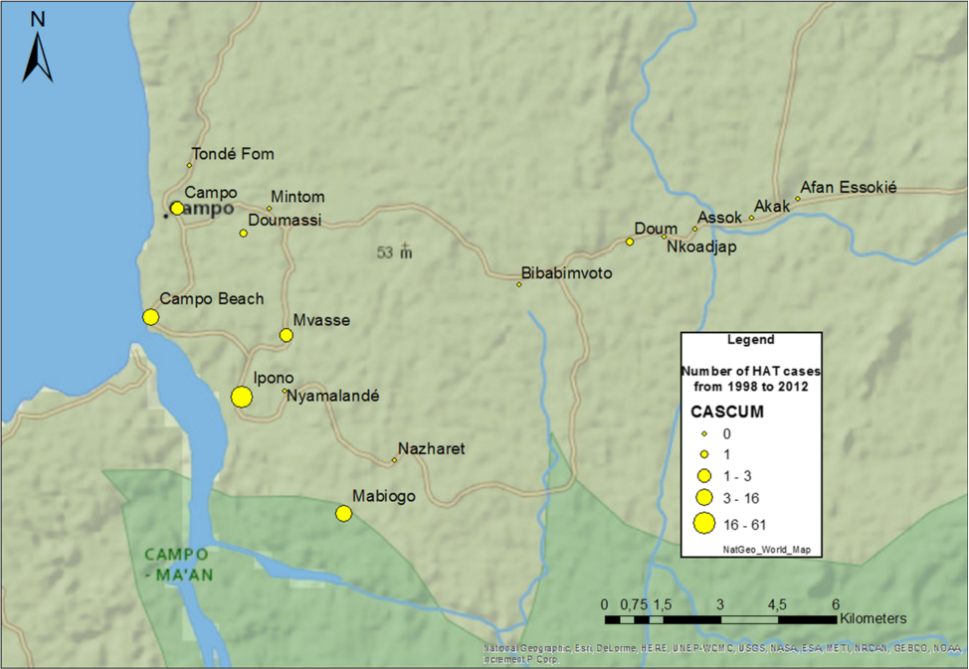
Number of HAT cases diagnosed in Campo from 1998 to 2012

Cameroon’s National Control Program (NCP) for sleeping sickness was created in 1998, although the first medical survey in Campo did not occur until 2002. Since then, the NCP regularly came every year to diagnose cases up until 2011, with the exception of 2005 (Fig. 1). In 2012, two cases were passively diagnosed in the Campo Hospital.

Between 2001 and 2012, 61 HAT cases were diagnosed in Ipono, a small village with approximately 300 inhabitants, which constitutes the epicenter of the HAT focus in the subdivision of Campo. All other cases were from Mabiogo (16), Campo Beach (15), and Campo (3). Aside from Ipono, five other cases were diagnosed in Mvasse, Doumassi (a small village near the entrance to Campo), and Bouandjo (on the road to Kribi). Taking into account these results, we observed the distribution of entomological results in this area.

### Entomological study

An entomological study was conducted during the 2012 dry season in the five villages most affected by the disease or tsetse flies: Campo, Campo Beach, Ipono, Mabiogo and Akak. Tsetse flies were captured for 3 days in pyramidal traps distributed in 162 capture points spread across the five sites, in an area of 295 km^2^. All points were georeferenced and the environmental characteristics of each trapping site such as for instance water point or river, forest, farmlands or human activity, tracks and houses were recorded. Tsetse flies were collected twice per day, and ADT (Apparent Density per day and per Trap) was calculated using the following formula: ADT = C/TD; where C is the number of flies caught, T the number of traps deployed and D the number of days of trapping. Subsequently, tsetse flies were identified at the species level using a binocular loop and it was determined whether they were in a teneral state or not, by thorax palpation, confirmed by dissection and presence of the residual bag at the beginning of the hindgut. Finally, flies were dissected to isolate the midgut for further molecular analysis. Dissecting tools were systematically dipped in a sodium hydroxide solution and rinsed in water between each dissection. Midguts were then stored in 70 % ethanol at ambient temperature.

### Molecular analysis

All samples collected from the field were incubated at minus 80 °C for ethanol evaporation. DNA was extracted using the Chelex method [20]. After extraction, DNA was stored at -20 °C until PCR analysis.

Trypanosomes were detected in midgut samples by PCR carried out as described by Tchouomene et al. [19] using primers TBR1 and TBR2 for the subgenus *Trypanozoon* [21]; TCF1 and TCF2 for *T. congolense*, forest-type [22]; TCS1 and TCS2 for *T. congolense*, savannah-type [21]; TVW1 and TVW2 for *T. vivax* and TS1 and TS2 for *T. simiae* [22]. All samples that were positive for TBR were subsequently amplified using TRBPA primers [23], in order to accurately identify *T. b. gambiense* to the subspecies level.

We used a heteroduplex PCR-based assay [24] to identify the feeding hosts of tsetse flies. The cytochrome B gene was amplified with primers specific to the vertebrate cytochrome B gene. Next, amplified fragments were hybridized with a driver, in this case from the *Cricetomys gambianus* (African giant pouched rat) cytochrome B DNA. Fragments were then migrated in a 5 % acrylamide/urea gel, and profiles were compared to references from humans or domestic mammals.

### Transmission risk index (TRI)

The transmission risk index was developed on the basis of most important factors characterizing the transmission of HAT notably the teneral flies, humans/tsetse fly contacts, the number of traps, the days of capture and the tsetse flies caught. Used to identify areas where vector control could be most effective at minimal cost, this index was originally developed by Laveissière et al. [25] and subsequently simplified by Laveissière and Grébaut [26]

$$ r=104\left(t+1\right)1.23\ast {n}^2\ast \left(\frac{C^{0.46}}{T{D}^{3.69}}\right) $$

This calculation takes into account the number of teneral flies (t), human blood meals (n), caught flies (C), traps (T), and collecting days (D); all these elements are considered as essential to offer tsetse flies the ability in transmitting *T. b. gambiense*. This index is calculated for sites (e.g. villages) including several capture traps. Nevertheless, if the use of this index can help in identifying places at risk, the fact that it does not integrate the parasite factor cannot make us consider it as a real transmission risk index but rather as a transmission capacity index.

### Risk gradient

This risk gradient was previously used [18] during an entomological study on transmission in Kinshasa (Congo) suburbs. This gradient is defined by seven levels of risk:

- RISK0: tsetse flies not present;
- RISK1: at least one tsetse fly;
- RISK2: at least one teneral fly or one human blood meal;
- RISK3: one teneral fly and one human blood meal;
- RISK4: presence of *T. b. gambiense* in the midgut;
- RISK5: RISK4+ RISK2;
- RISK6: RISK4 + RISK3.

### Geographical information system

Mapping material for this region of Cameroon was provided by ESRI. Projections of all epidemiological and entomological results were performed using ArcGis®.

## Results

Out of the 991 tsetse flies caught in traps, 96.6 % (957) were identified as *Glossina palpalis palpalis*. The remaining 3.4 % were *Glossina pallicera* (29), *Glossina caliginea* (1), *Glossina tabaniformis* (3) and *Glossina nigrofusca* (1). The teneral stage accounted for 5 % of the flies.

### Vector distribution

**Fig. 2:**
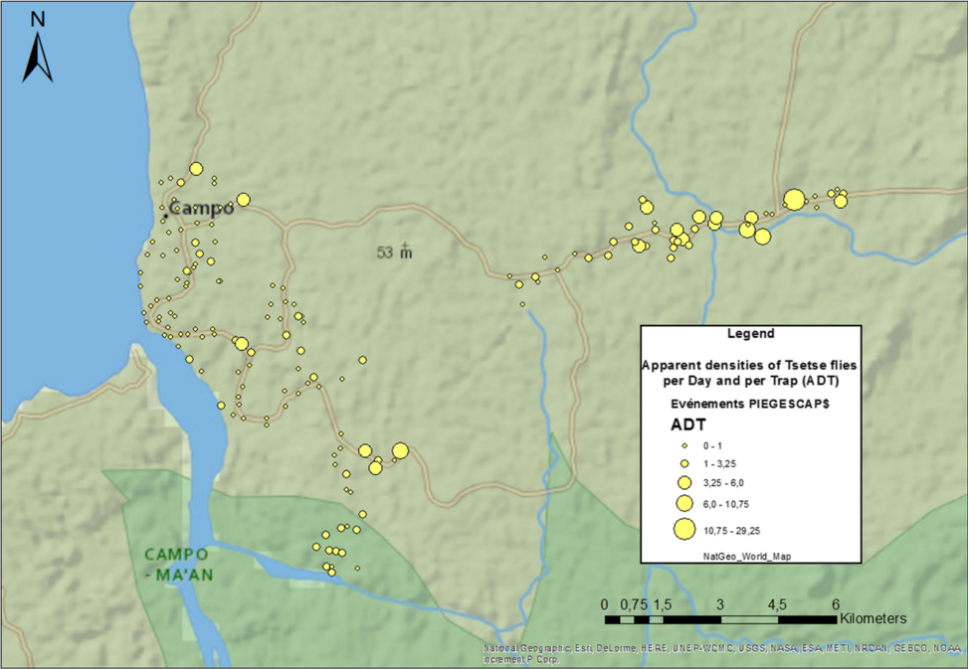
Map of the distribution of the apparent density of tsetse flies per day and per trap (ADT) during the 2012 dry season in Campo

Campo Beach, located at the western end of our study area, yielded the lowest observed ADT (0.2) in this study (Fig. 2). Surrounding Campo, the most populous community in the area, the ADT was observed to be 1.7. The ADTs were larger in value in forest communities, including Mabiogo and all the villages along the eastern axis; it reached an apex of 9.2 in Akak, which constitutes the greatest density observed in the area. Overall, the average ADT score was 1.5.

We also used a classification scheme to divide ADT scores into 3 groupings: ADT1 represents 0 < ADT ≤1 fly, ADT2 represents all traps yielding 1 < ADT ≤ 5 flies and ADT3 represents an ADT > 5 flies. According to this scheme, ADT1 was significantly present in Mvasse (Chi-square test: *χ*^2^ = 6.891, *df* = 1, *P* = 0.0009). ADT3 was significantly present along the riversides of the secondary water system (Chi-square test: *χ*^2^ = 6.954, *df* = 1, *P* = 0.008),

### Blood meal analysis

Out of the 991 captured tsetse flies, 962 (97 %) were dissected and 143 blood meals were analyzed by the heteroduplex PCR-based assay. Out of these, the cytochrome B gene could be amplified in 76 and the origin of 57 (75 %) of these latter could be determined. We considered that among the 143 isolated bloodmeals, the cytochrome B gene of 53 % (67/143) of them was not amplified due to an insufficient quantity of blood or to an advanced digestive process. Among the identified ones, Human is the major host in the area, representing 60.2 % (46/76) of all analyzed blood meals. Human blood meals were significantly more numerous in the villages in the epicenter than in those along eastern axis (Chi-square test: *χ*^2^ = 21.283, *df* = 1, *P* < 0.0001). The other identified blood meals were from pig (7.9 %), sheep (2.6 %), goat (1.3 %) and sitatunga (2.6 %). The reminder (25 %) was supposed to be from other unidentified wild animals.

### Trypanosome infections

PCR analysis identified 28 midguts as infected, yielding a 19.6 % infection rate. The most frequently found infection was from *T. congolense* forest-type (17/28), followed by *T. congolense* savannah-type (7/28). Five of these infected midguts were of a mixed infection type (e.g. TBB/TCF or TBB/TCS). One fly was found infected with *T. simiae* next to a pigsty in Campo. Three samples were infected with *T. brucei* (*s.l.*), including 1 infected by *T. b. gambiense*.

### Transmission risk index

The TRI ranged from 0–700, according to which village the index was applied to (Table 1). The score was particularly high in some villages along the eastern axis (e.g. an index of 700 in Akak). By contrast, the scores were not high in the villages at the epicenter of the focus. In Campo, this risk translates into a close contact between the human population and tsetse flies.

**Table 1:** Entomological results in the Campo focus in dry season 2012

Villages	GLO (n)	GPP (n)	PAL (n)	CAL (n)	TAB (n)	FUS (n)	TEN (n)	DISS (n)	TRAPS (n)	ADT	TRI
Campo	141	141	0	0	0	0	7	141	27	1.2	7
Campo Beach	16	16	0	0	0	0	0	16	33	0.1	0
Mabiogo	182	178	4	0	0	0	4	182	25	1.8	2
Ipono	53	52	1	0	0	0	1	53	12	1.1	0
Mvasse	61	59	2	0	0	0	0	61	21	0.7	0
Akak	220	212	4	1	3	0	12	213	8	6.9	770
AfanEssokié	39	35	4	0	0	0	0	30	7	1.4	2
Doum	117	107	10	0	0	0	8	112	10	2.9	56
Nkoadjap	142	137	4	0	0	1	14	134	13	2.7	5
Nazareth	20	20	0	0	0	0	2	20	6	0.8	0
Total	991	957	29	1	3	1	48	962	162	2.0*	84**

*GLO* glossinas captured; *GPP* *G. palpalispalpalis*; *PAL* *G. pallicera*; *Cal* *G. caliginea*; *TAB* *G. tabaniformis*; *FUS* *G. nigrofusca*; *TEN* teneral flies; *DISS* Glossinas dissected; *ADT* apparent density per day and per Trap; *TRI* transmission risk index; *average ADT; **average TRI

### Risk gradient

**Fig. 3:**
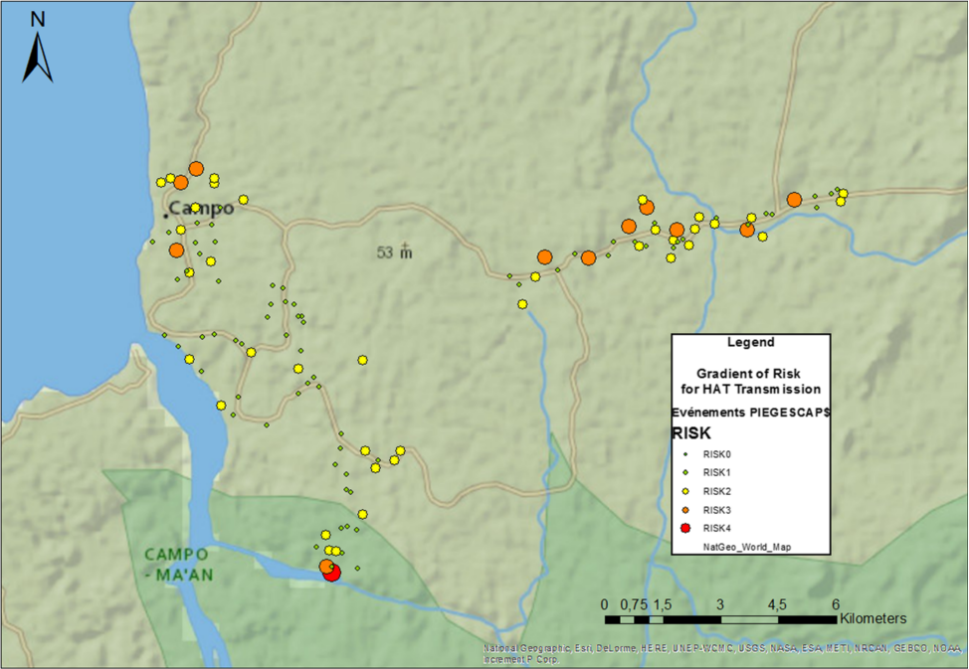
Risk gradient for HAT transmission in the Campo focus

The risk gradient was applied to each capture point, which enabled plotting a distribution map of this gradient (Fig. 3). The RISK5 and RISK6 levels were not observed. As for TRI, and when compared to the villages of the epicenter, there was a significant accumulation of places at RISK3 along the eastern road (Chi-square test: *χ*^2^ = 6.824, *df* = 1, *P* = 0.009), which could favor an endemic situation if a reservoir were to be introduced in this area. The RISK3 level was also associated with several locations surrounding Campo city. Mabiogo, a village in the epicenter, offers the highest-level risk gradient (RISK4) in the area.

These results show that xenomonitoring via entomological observations provides essential information regarding the risk of humans’ exposure to tsetse bites, where vector control operations must be performed in this HAT focus. Next, we will discuss how this focus could still be active, despite the regular screening conducted by the Cameroonian NCP.

## Discussion

We have conducted a study to inventory the sleeping sickness vectors and trypanosomes within the Campo area. The results focus on the apparent density of tsetse fly populations, as well as their infections with trypanosomes. Combining this information with the identification of host blood sources has allowed us to target locations at risk in this area. Furthermore, our xenomonitoring approach can facilitate vector control and epidemiological surveillance recommendations. Nevertheless, the absence of geo-referenced traps and the potential differences in climatic conditions during past studies obliges us to be cautious when comparing our study with previous results.

*Glossina palpalis palpalis* is the predominant species of *Glossina* in the Campo area, representing 96.6 % of tsetse flies. Nevertheless, and for the first time since Eouzan’s unpublished study (1974), *Glossina tabaniformis* were captured and identified in the area. This reappearance of *G. tabaniformis* mainly occurred in the traps located at the eastern side of the study area, a few kilometers from the Campo Ma’an National Park. A hunting ban as well as the presence of the National Park probably favors the increased numbers of wild animals that are preferential feeding hosts for tsetse flies of the *fusca* group. During our study we also captured 29 *Glossina pallicera* (2.9 % of captured flies), whereas Mbida Mbida [27] reported 25.1 % at the same period of the year. In 1998, Morlais et al. [7] reported that *Glossina palpalis* made up 81 % of the captured flies, although Mbida Mbida estimated their representation to be 61.8 % at the same season we performed our study. Our result could indicate that the proportion of other tsetse flies species is decreasing in the Campo area, with the exception of *Glossina tabaniformis*.

The lowest ADT scores (< 1) were observed in Mvasse, located at the Ntem river mouth and the beginning of the coastal plain, in the epicentre of the focus. Regarding ADT distribution, tsetse fly densities decline as sampling moves in closer to the coast; this phenomenon has already been described in the majority of our entomological studies performed in the area. However, the numbers of tsetse flies are up to 30 times greater on the eastern axis, where there are almost no HAT cases. This suggests that HAT transmission does not depend exclusively on the vector’s density neither on the risk of humans’ exposure to the bite of competent flies, but also depends on the existence of a parasite reservoir. For instance, the existence of a reservoir in the Eastern area of the study zone could have been translated in a real endemic situation in the villages of the eastern axis, but this was not the case although the risk of humans’ exposure was high. This makes us conclude that there is no active reservoir in the eastern axis.

In order to isolate the midgut under optimal conditions for laboratory analyses, we limited the number of traps to 50 per circuit and collected twice per day during 3 days of capture to obtain enough flies. After dissection, each midgut was isolated in a 70 % ethanol solution to preserve DNA from degradation. Subsequently, we identified trypanosomes using molecular markers. Using this approach, we were able to dissect 97 % of captured flies and to diagnose a 19.6 % infection rate by trypanosomes. This rate is similar to the 16.16 % infection rate determined by Morlais et al. [7] in the Campo area. In our study, *Trypanosoma brucei* (*s.l.*) was only present in two infected midguts. In contrast, Farikou et al. [15] determined a 31.8 % (22/132) infection rate, as well as a 16.7 % infection rate for *T. b. brucei*; however, it should be noted that these results were obtained with the isolates from three 4-day long vector trapping campaigns. In the present study, the trypanosome subgenus *Nannomonas* makes up the majority of infections in the area (25/28), including *T. congolense* forest and savannah types, and *T. simiae*. The identification of *T. simiae* next to a pigsty in Campo represents a real danger, due to the high pathogenicity of *T. simiae* for pigs [28]. Similarly, Nimpaye et al. [29] determined a very low infection rate (0.64 %) in Campo. Attention should be given by veterinary services to trypanosome infections of domestic animals in this area; vector control tools such as traps or tiny targets could be used to protect agricultural activity that is in full expansion in the area.

One sample from Mabiogo was positive for *T. b. gambiense*, yielding a prevalence of 0.1 % in tsetse flies, and the evidence of an active reservoir in the epicenter of the focus. Based on this low vector prevalence, we decided against dissecting and isolating salivary glands as a means to investigate vector competence. The identification of *T. b. gambiense* in tsetse fly is an important argument in favor for the deploying of vector control tools like traps and “tiny targets” in this area.

Analysis of the 143 blood meals in this study supports that the main host in the area is man accounting for 60.2 % of the analyzed blood meals. Pigs represent the second most identified host in the area, accounting for 7.9 % of the blood meals. The latter result is similar to the 6.8 % infection rate reported by Njiokou et al. [30] using the same method of heteroduplex PCR-based analysis of cytochrome B. Farikou et al. [15] succeeded in identifying up to 22.7 % of blood meals from pigs and 62.9 % from humans, using both heteroduplex and sequencing methods. In the present study, a large number of blood meals were not adequately fresh, thus creating sensitivity problems. As a result, the quantity of amplified products was not enough for the hybridization reactions that enable the identification of blood meals’ origins. Despite the low ADT score, human blood meals were significantly more numerous in the villages in the epicenter than in those along eastern axis, indicating that transmission does not necessarily depend on fly density, but on other factors such as for instance the human exposure and the presence of *T. b. gambiense*.

The transmission risk index corroborated the results obtained when applying the risk gradient. Paradoxically, all of the conditions for an active transmission are concentrated along the eastern axis of the zone where the HAT cases are rare, while a medium risk level is observed in Campo and a lower risk is localized in the villages of the epicenter of the focus. TRI expresses the importance of the contact between humans and tsetse flies, and can be compared to RISK2 and 3. RISK3 indicates that all conditions for transmission are assembled for pathogen propagation, in the event that a reservoir is introduced into these areas. At this level, *T. b. gambiense* infections were not taken into consideration since this parasite was supposed to circulate in all active HAT foci like the Campo focus. However, in the current context where the disease prevalence is very low and where the transmission occurs probably in specific areas or villages, the use of this TRI seems to be not really relevant because their values were high in villages surrounding the epicenter, where no infection due to *T. b. gambiense* was identified. In such context, the spatial distribution of risk gradient provides data showing not only where the transmission occurs, but also biotopes and villages where control operations must be focalized. The non-identification of *T. b. gambiense* in tsetse flies of villages surrounding the epicenter and its identification in one village of the epicenter indicates that *T. b. gambiense* infections are an important component that must be taken into account for the estimation of HAT transmission. This observation is in line with those previously reported by Njitchouang et al. [31] who developed a new transmission index by taking into account the infections due to *T. b. gambiense* as well as blood meals taken on animals. In this study, the determination of just one fly infected with *T. b. gambiense* did not allow us to apply this new index. The recent mathematical projections made by Funk et al. [32], Stone & Chitnis [33] and Rock et al. [34] would be worth being applied in the Campo area to evaluate the importance of the animal reservoir. Nevertheless, the TRI reported in this study show at least areas and villages of potential high transmission risk where control operations could subsequently be targeted if *T. b. gambiense* is introduced in villages surrounding the epicenter.

This level of risk is observed around Campo and along the eastern axis of Akak, particularly in collective water points. Twenty-two collective water points have been identified across the whole area, which should constitute prime targets for vector control. The presence of several pigsties in Campo as well as a few cases of sleeping sickness could amplify an endemic situation, knowing pigs as a potential reservoir [31]. Such events can occur through the important socioeconomic mutations reported in the Campo area [35].

## Conclusion

The diagnosis of seven new cases in 2014 (Ebo’o Eyenga, personal communication) demonstrates that the sleeping sickness focus of Campo is still active. Our entomological results enabled us to identify locations that favor transmission. In the near future, the arrival of several thousands of new inhabitants in the region, due to the creation of a local deep-water harbor and the restoration of forestry exploitation, will expose the people to the risk of transmission. This risk will be amplified by the need of the population to develop agricultural productions for food, including fields and small ruminant breeding, all of which will promote contact with tsetse flies and contribute to the expansion of the disease [34]. The deployment of traps and targets and the involvement of veterinary services (screening and treatment of domestic animals against trypanosomosis) are two possible solutions that must complement classical mass screening and treatment of humans for the elimination of HAT in the Campo focus. Regarding the complexity of the hydrographic network, the eradication of tsetse flies should be too difficult to accomplish. Xenomonitoring of tsetse flies provides new hope in that it offers a way to optimize a vector control strategy that can efficiently protect the population from HAT transmission, complementing classical public health approaches. Finally, financial efforts can be made by international organizations, private funds and the Cameroonian government to support economic development, to contribute efficiently to the elimination of sleeping sickness from this area beginning to enter a changing phase.
